# Dynamics of NK, CD8 and Tfh cell mediated the production of cytokines and antiviral antibodies in Chinese patients with moderate COVID‐19

**DOI:** 10.1111/jcmm.16044

**Published:** 2020-11-03

**Authors:** Lin Yan, Bei Cai, Yi Li, Min‐Jin Wang, Yun‐Fei An, Rong Deng, Dong‐Dong Li, Li‐Chun Wang, Huan Xu, Xue‐Dan Gao, Lan‐Lan Wang

**Affiliations:** ^1^ Department of Laboratory Medicine West China Hospital Sichuan University Chengdu China; ^2^ Center of Infectious Diseases West China Hospital Sichuan University Chengdu China

**Keywords:** CD8, COVID‐19, NK, Tfh, TIM‐3

## Abstract

Recent studies have demonstrated a marked decrease in peripheral lymphocyte levels in patients with coronavirus disease 2019 (COVID‐19) caused by severe acute respiratory syndrome coronavirus 2 (SARS‐CoV‐2). Few studies have focused on the changes of NK, T‐ and B‐cell subsets, inflammatory cytokines and virus‐specific antibodies in patients with moderate COVID‐19. A total of 11 RT‐PCR‐confirmed convalescent patients with COVID‐19 and 11 patients with non‐SARS‐CoV‐2 pneumonia (control patients) were enrolled in this study. NK, CD8^+^ T, CD4^+^ T, Tfh‐like and B‐cell subsets were analysed using flow cytometry. Cytokines and SARS‐CoV‐2‐specific antibodies were analysed using an electrochemiluminescence immunoassay. NK cell counts were significantly higher in patients with COVID‐19 than in control patients (*P* = 0.017). Effector memory CD8^+^ T‐cell counts significantly increased in patients with COVID‐19 during a convalescent period of 1 week (*P* = 0.041). TIM‐3^+^ Tfh‐like cell and CD226^+^ Tfh‐like cell counts significantly increased (*P* = 0.027) and decreased (*P* = 0.022), respectively, during the same period. Moreover, ICOS^+^ Tfh‐like cell counts tended to decrease (*P* = 0.074). No abnormal increase in cytokine levels was observed. The high expression of NK cells is important in innate immune response against SARS‐CoV‐2. The increase in effector memory CD8^+^ T‐cell counts, the up‐regulation of inhibitory molecules and the down‐regulation of active molecules on CD4^+^ T cells and Tfh‐like cells in patients with COVID‐19 would benefit the maintenance of balanced cellular and humoural immune responses, may prevent the development of severe cases and contribute to the recovery of patients with COVID‐19.

## 1 INTRODUCTION

The outbreak of coronavirus disease 2019 (COVID‐19), which is caused by severe acute respiratory syndrome coronavirus 2 (SARS‐CoV‐2), is an urgent threat to global health. As it is a pandemic, research on its clinical characteristics, treatment, immune response and vaccine development is urged to be conducted.

Natural killer (NK) cells, CD8^+^ T, CD4^+^ T and B lymphocytes are essential in antiviral immune response. In a study, the number of NK, T and B cells in various types of viral infection has been reported to be different, indicating a potential correlation between these differences and viral pathogenic mechanisms.^1^ In other studies, the dysregulation of immune response was observed in patients with severe COVID‐19.^2^, ^3^ Moreover, a multicenter retrospective study revealed that lower lymphocyte count was an independent high‐risk factor associated with COVID‐19 progression.^4^ Furthermore, an increase in the number of peripheral lymphocyte subset in patients with COVID‐19 was associated with improved clinical symptoms and treatment efficacy.^3^, ^5^ In patients with severe COVID‐19, lymphopenia resulted from drastically reduced numbers of NK, CD8^+^ T, CD4^+^ T and B cells, but not in patients with mild or moderate disease^2^, ^6^, ^7^, ^8^; these studies mainly focused on the correlation between lymphocyte count and COVID‐19 severity, as no results on the change in the number of NK cells and lymphocyte subsets in patients with moderate COVID‐19 were reported. Additionally, it remains unknown whether there were differences in these cells between patients with moderate COVID‐19 and those with non‐SARS‐CoV‐2 infectious pneumonia. Therefore, elucidating the dynamic characteristics of NK cells and multiple lymphocyte subsets in patients with moderate COVID‐19 during recovery period would help us better understand proper immune response against SARS‐CoV‐2 infection; such understanding may be utilized for the selection and development of therapeutic drugs.

Infection with SARS‐CoV‐2 can activate innate and adaptive immune responses. NK cells participate in the elimination of virus‐infected cells without viral antigen presentation.^9^ Cytotoxic T lymphocyte (CTL), an activated subset of CD8^+^ T cells, can kill virus‐infected cells by releasing perforin.^10^ CD4^+^ T cells, particularly T follicular help (Tfh) cells, can promote the production of virus‐specific antibodies by activating Tfh‐dependent B cells.^11^ Moreover, peripheral Tfh‐like cells were defined as CXCR5^+^CD4^+^ T cells.^12^ After viral infection, the activated immune system would not only directly induce antiviral cellular and humoural immune responses but also develop memory CD4^+^ and CD8^+^ T cell subsets as a preparation for secondary infection.^13^ B cells differentiate from naïve or transitional to mature cells and finally differentiate into plasma cells, which produce virus‐specific antibodies. In addition to the detection of SARS‐CoV‐2‐specific antibodies for the diagnosis of COVID‐19,^14^ these specific antibodies may neutralize SARS‐CoV‐2. Furthermore, we analysed other biomolecules, including inhibitory cell surface molecules: T‐cell immunoglobulin and mucin 3 (TIM‐3), programmed cell death‐1 (PD‐1), T‐cell immunoglobulin and ITIM domain (TIGIT), and active cell surface molecules: inducible costimulatory molecule (ICOS) and CD226, which could bind to CD155 on antigen‐presenting cells (APC) and compete with TIGIT.^15^, ^16^ The serum inflammatory cytokine levels in patients with severe COVID‐19 were observed to be abnormally increased and were used to predict the risk of cytokine storm.^17^, ^18^ Furthermore, serum interleukin (IL)‐6, IL‐1β, IL‐8, IL‐10, tumour necrosis factor (TNF)‐α and C‐reactive protein (CRP) levels were analysed to determine the variability of lymphocyte subsets in the present study.

Although patients with severe COVID‐19 have high mortality, most patients with the disease exhibit only mild to moderate symptoms.^6^, ^7^ Given that SARS‐CoV‐2 is not a well‐known virus, the host resistance of virus‐infected patients is a key factor in determining the success or failure of host recovery. The analyses of multiple NK, CD8^+^ T, CD4^+^ T and B cell subsets, the production of virus‐specific antibodies, the expression of associated‐activation and exhaustion molecules, the secretion of inflammatory cytokines in patients with moderate COVID‐19 during convalescent period, and the identification of the differences in the cell subsets between SARS‐CoV‐2 and other pathogenic microorganism (non‐SARS‐CoV‐2) infection would provide useful information on immune response against SARS‐CoV‐2.

## 2 METHODS

### 2.1 Patients

A total of 11 patients with COVID‐19 (admitted from 31 January to 9 February 2020) and 11 patients with non‐SARS‐CoV‐2 pneumonia (control patients) from West China Hospital of Sichuan University were enrolled. The latest follow‐up assessment was until 19 March 2020. A confirmed COVID‐19 case was defined as (a) positive for SARS‐CoV‐2 RNA in the real‐time reverse‐transcriptase polymerase chain reaction (RT‐PCR) assay of nasal or pharyngeal swab, sputum, stool specimens as described by the WHO; (b) negative for 11 common respiratory pathogens tested by using RT‐PCR. Patients with non‐SARS‐CoV‐2 pneumonia were enrolled based on the following criteria: (a) the nasopharyngeal swab, sputum or stool specimens from the patients were negative in SARS‐CoV‐2 RNA detection; and (b) the diagnosis of pneumonia was based on symptoms and chest computed tomography (CT) results without culture‐confirmed pathogens; or (c) pathogenic evidence of bacteria, fungus or virus from nasopharyngeal swab, sputum or blood specimen. Moreover, patients with COVID‐19 and the control patients were tested for 11 common respiratory pathogens by using RT‐PCR; the 11 pathogens are human influenza A virus, influenza B virus, Mycoplasma pneumoniae, Chlamydia, parainfluenza virus, adenovirus, bocavirus, rhinovirus, metapneumovirus, coronavirus and respiratory syncytial virus.

NK cell, T lymphocyte and B lymphocyte subset phenotyping and counting were performed on February 17 and February 25, respectively. The 11 patients with COVID‐19 and 11 patients with non‐SARS‐CoV‐2 pneumonia received a first phenotyping test on 17 February 2020. Subsequently, only 10 patients with COVID‐19 received a second phenotyping test on 25 February 2020, as one patient had been discharged at the time. All patients with COVID‐19 were administered Kaletra as an antiviral therapy and given the corresponding symptomatic treatment upon the diagnosis of COVID‐19. Arbidol (or ribavirin) was administered in patients with persistent SARS‐CoV‐2 RNA. The patients whose nasopharyngeal swab tested positive for SARS‐CoV‐2 were administered interferon‐α through atomization. Antibiotics were used in patients with COVID‐19, when a combination of bacterial infections was identified. Patients with non‐SARS‐CoV‐2 pneumonia were administered moxifloxacin or levofloxacin. The design of this study was approved by the Ethics Committee of West China Hospital, and all participating patients provided written informed consent prior to enrolment.

### 2.2 Data collection

Epidemiological history, clinical symptoms, laboratory data, chest CT results and treatment regimen records were collected from the electronic hospital and laboratory information system. To verify data accuracy, the medical records of the patients were independently reviewed by two researchers (XH and GXD). The severity of COVID‐19 was defined according to the revised trial version of COVID‐19 Diagnosis and Treatment Guidance.^19^ A patient with a mild COVID‐19 was defined as a patient with mild clinical symptoms and no pneumonia upon chest CT imaging. A patient with moderate COVID‐19 was defined as that with symptoms, including fever, respiratory symptoms and pneumonia upon chest CT imaging. The criteria for severe cases included at least one of the following: (a) shortness of breath and a respiratory rate of ≥30 times/min; (b) pulse oximeter oxygen saturation (SpO_2_) ≤93% at rest; (c) ratio of partial pressure of arterial oxygen (PaO_2_) to the fraction of inspired oxygen (FiO_2_) ≤300 mm Hg; and (d) the clinical symptoms must have had progressively aggravated, and chest CT images must have indicated that the lesion had progressed significantly to >50% within 24‐48 hours. The severity of non‐SARS‐CoV‐2 pneumonia in patients was based on the severity standard of patients with COVID‐19. Furthermore, the patients who exhibited clinical response were defined as patients with COVID‐19 in the convalescent period and defined according to the following criteria: (a) symptom alleviation (eg, fever, cough) must be observed; and (b) improvement of radiological abnormalities of chest CT results compared between the first and second phenotyping test.

### 2.3 Phenotyping

To determine the NK cells and lymphocyte subsets in patients, heparin‐anticoagulated whole blood samples were collected and stained with (1) CD3‐PerCP (BD Biosciences, San Jose, CA,), CD4‐BV510 (BD Biosciences, San Jose, CA), CD8‐APC (BD Biosciences, San Jose, CA), CD45RO‐BV421 (BioLegend, San Diego, CA), and CCR7‐PE (BD Biosciences, San Jose, CA); (2) CD4‐APC‐Cy7 (eBioscience, San Diego, CA), CXCR5‐APC (eBioscience, San Diego, CA), TIM‐3‐PerCP (eBioscience, San Diego, CA), TIGIT‐FITC (eBioscience, San Diego, CA), CD226‐PE‐Cy7 (eBioscience, San Diego, CA), PD‐1‐BV510 (BD Biosciences, San Jose, CA), and ICOS‐BV421 (BD Biosciences, San Jose, CA); (3) CD19‐PE (eBioscience, San Diego, CA), CD27‐PE‐Cy7 (eBioscience, San Diego, CA), CD38‐APC (eBioscience, San Diego, CA), CD24‐APC‐Cy7 (eBioscience, San Diego, CA), IgM‐BV421 (eBioscience, San Diego, CA,), and IgD‐FITC (eBioscience, San Diego, CA); and (4) CD3‐FITC (BD Biosciences, San Jose, CA) and CD16 + CD56‐PE (BD Biosciences, San Jose, CA). Cell counting was performed with absolute counting tubes with a certain number of beads (BD Biosciences, San Jose, CA). The samples were measured using FACS Canto II (BD Biosciences, San Jose, CA). Gating analysis was performed with FlowJo V10 (BD Biosciences, San Jose, CA).

### 2.4 Measurement of cytokines, inflammatory factors and SARS‐CoV‐2‐specific antibodies

Serum was collected for the analyses of cytokines, inflammatory factors and SARS‐CoV‐2‐specific IgM/IgG antibodies. IL‐6 levels were measured using an electrochemiluminescence immunoassay (Roche Diagnostics, Rotkreuz, Zug, Switzerland). IL‐1β, IL‐2R, IL‐8, IL‐10 and TNF‐α were measured using chemiluminescence analysis (Siemens, Erlangen, Bavaria). CRP was measured using a scattering immunoturbidimetric assay (Beckman Coulter, Indianapolis, IN). SARS‐CoV‐2‐specific IgM/IgG antibodies were measured using chemiluminescence analysis (YHLO Biotech Co., Shenzhen, Guangdong).

### 2.5 Statistics

Continuous and categorical variables are presented as median (range) and number (%), respectively. Mann‐Whitney *U* test was used to compare the differences among percentages and counts obtained from the phenotyping of different groups. Chi‐square test and Mann‐Whitney *U* test were used to compare the categorical variables among different groups. A paired nonparametric test was performed to compare changes in the number of immune cells in the first and second phenotyping tests. Statistical analysis was performed with SPSS, version 21.0 (IBM Corp., Sterling Forest, NY), and graphic representations were generated using GraphPad Prism version 5.01 (GraphPad Software, San Diego, CA).

## 3 RESULTS

### 3.1 Demographic data and clinical characteristics of patients with moderate COVID‐19 and control patients

**FIGURE 1:**
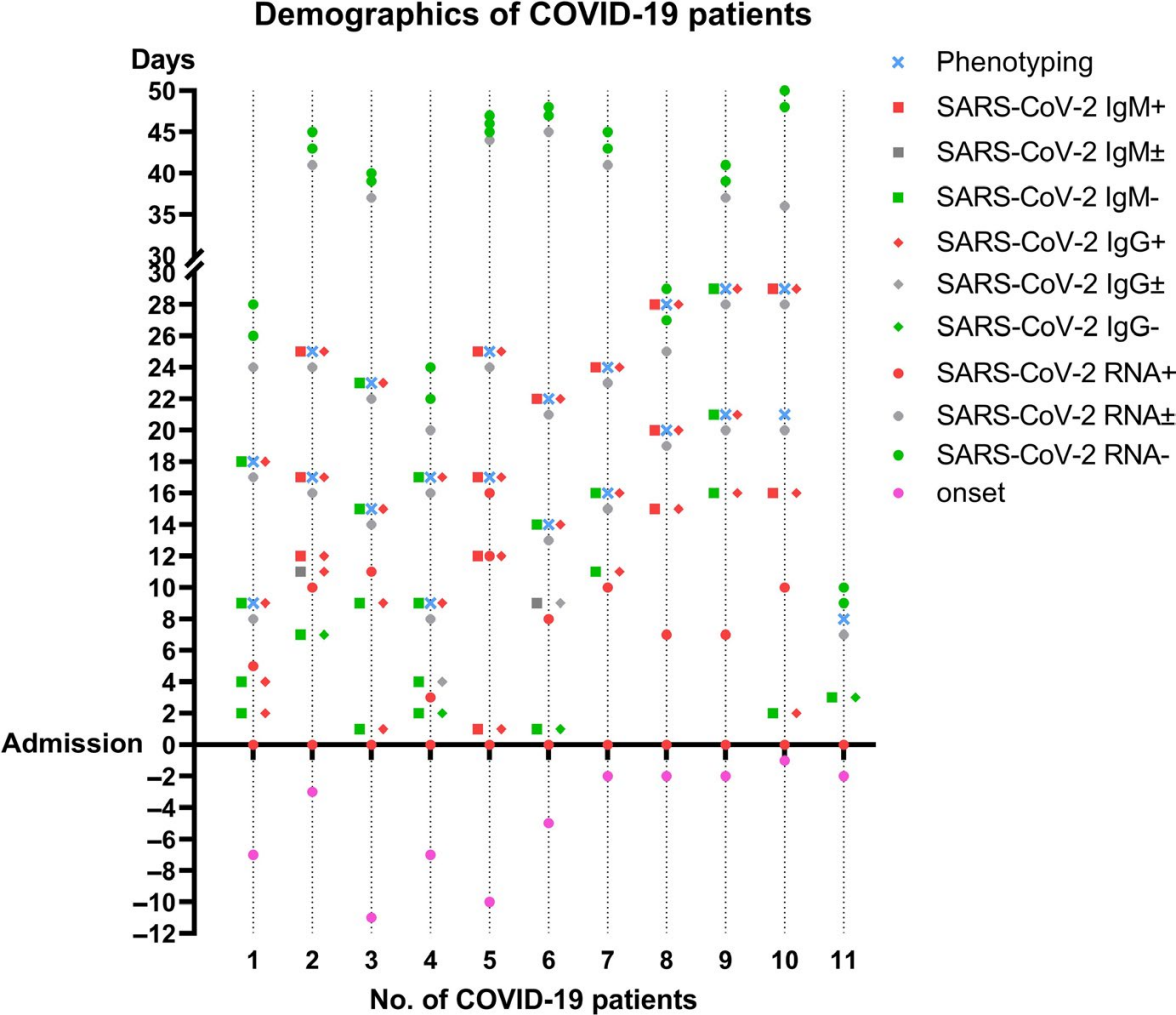
Demographics of patients with COVID‐19. Admission: date of admission in West China Hospital; rose red circles indicate the onset of symptoms; green, grey and red circles indicate negative, suspected and positive results, respectively, for SARS‐CoV‐2 RNA testing; green, grey and red diamonds indicate negative, suspected and positive results, respectively, for SARS‐CoV‐2 IgG antibodies; green, grey and red squares indicate negative, suspected and positive results, respectively, for SARS‐CoV‐2 IgM antibodies. Blue crosses indicate phenotyping tests

The demographic data and clinical characteristics of patients with moderate COVID‐19 who were enrolled are listed in Table 1 and Figure 1. The clinical symptoms of patients with COVID‐19 were consistent with those of patients in previous studies^20^, ^21^: fever (7/11, 63.6%), cough (9/11, 81.8%), sputum (4/11, 36.4%), fatigue (2/11, 18.2%), stuffy nose (2/11, 18.2%), runny nose (1/11, 9.1%), chest tightness (3/11, 27.3%) and headache (1/11, 9.1%). Information regarding the results of SARS‐CoV‐2‐specific antibodies, the severity of disease, presence of pathogenic microorganisms, treatment regimens provided to the patients and clinical response is shown in Table 1.

**TABLE 1:** Demographics and clinical characteristics

	COVID‐19 (N = 11)	Control (N = 11)	*P*‐value
Age (y)	46 (19‐76)	33 (22‐63)	0.562
Gender, male/female (N)	5/6	5/6	1.000
Period from onset of symptoms to admission (d)	3 (1‐11)	4 (2‐20)	0.217
Hospital duration (d)	41 (10‐50)	7 (2‐18)	<0.001
From Wuhan (N)	5	0	—
Contacted people from Wuhan (N)	6	0	—
SARS‐CoV‐2‐specific antibodies			
Specific IgM positive, 1st/2nd (N)	4/6	0/–	—
Specific IgG positive, 1st/2nd (N)	10/10		
The severity of patients, N (percentage)			
Mild	0 (0%)	0 (0%)	—
Moderate	11 (100%)	11 (100%)	
Severe	0 (0%)	0 (0%)	
Pathogenic microorganism, N (percentage)			
SARS‐CoV‐2	11 (100%)		—
*Mycoplasma pneumoniae*		5 (45.5%)	
Unknown		6 (54.5%)	
Treatment regimen, N (percentage)			—
Antiviral	11 (100%)	0 (0%)	
Kaletra	10 (90.9%)		
Arbidol	10 (90.0%)		
Interferon‐α	4 (36.4%)		
Ribavirin			
Antibiotic	8 (72.7%)	11 (100%)	
Moxifloxacin	5 (45.5%)	9 (81.8%)	
Levofloxacin	1 (9.1%)	2 (18.2%)	
Ceftriaxone	2 (18.2%)		
Clinical response, N (percentages)	11 (100%)	—	—

Note

Data are presented as median (range) or numbers or percentages. COVID‐19: Coronavirus Disease 2019; Control: patients with non‐SARS‐CoV‐2 pneumonia; 1st/2nd: first phenotyping test/second phenotyping test; N: numbers.

### 3.2 NK cells and lymphocyte subsets in patients with COVID‐19 and control patients

**FIGURE 2:**
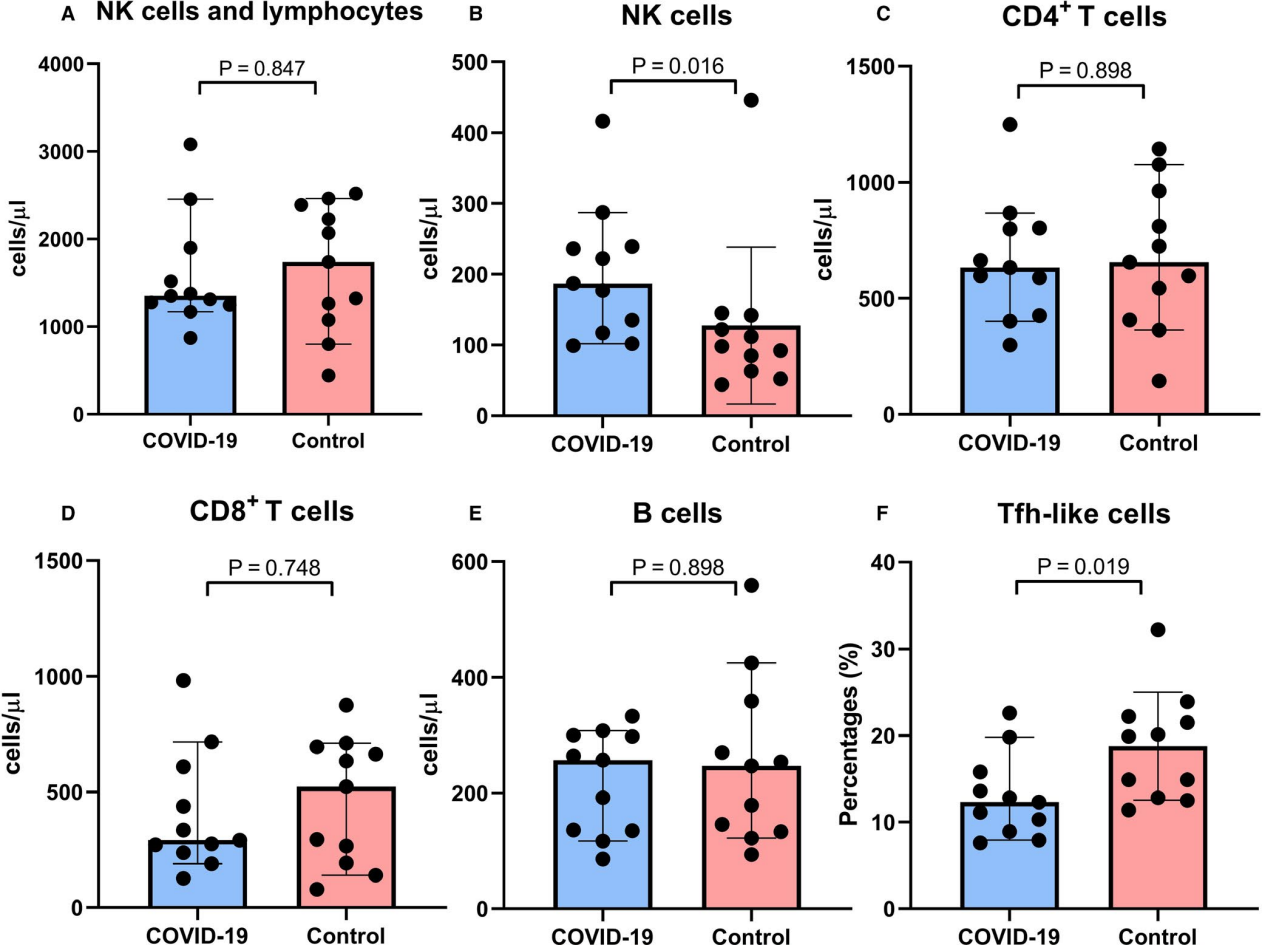
NK and lymphocyte subsets between COVID‐19 patients and control patients. Data are presented as median (95% confidence interval) and were analysed by using Mann‐Whitney *U* test. (A) Number of NK cells and lymphocytes (B) Number of NK (CD16^+^ or CD56^+^) cells (C) Number of CD4^+^CD3^+^ T cells (D) Number of CD8^+^CD3^+^ T cells (E) Number of B (CD19^+^) cells (F) percentage of CXCR5^+^CD4^+^ T cell in patients with COVID‐19 and control patients

No significant difference in the total number of NK cells and lymphocyte subsets, the absolute number (or percentage) of CD8^+^ T, CD4^+^ T or B cells was found between patients with moderate COVID‐19 and control patients (Figure 2A,C–E, Data S1). The absolute number of NK cells was significantly higher in patients with COVID‐19 than in the control patients (187 cells/μL [99‐416 cell/μL] vs 98 cells/μL [44‐446 cells/μL], *P* = 0.017, Figure 2B). The percentage of Tfh‐like cells was significantly lower in patients with COVID‐19 than in the control patients (12.30% [7.62%‐22.60%] vs 19.90% [11.40%‐32.20%], *P* = 0.021, Figure 2F). No significant differences in the absolute number or percentage of naïve CD4^+^ or CD8^+^, memory CD4^+^ or CD8^+^, TIM‐3^+^CD4^+^, TIGIT^+^CD4^+^, PD‐1^+^CD4^+^, ICOS^+^CD4^+^, CD226^+^CD4^+^ T cells, TIM‐3^+^, TIGIT^+^, PD‐1^+^, ICOS^+^, CD226^+^ Tfh‐like cells and B‐cell subsets at different differentiation stages were observed between the two groups (Data S1,S2). In summary, only NK cells and Tfh‐like cells were observed to be different between COVID‐19‐infected patients and the control patients.

### 3.3 Changes in the number of NK cells and lymphocyte subsets in patients with COVID‐19 during convalescent period

**FIGURE 3:**
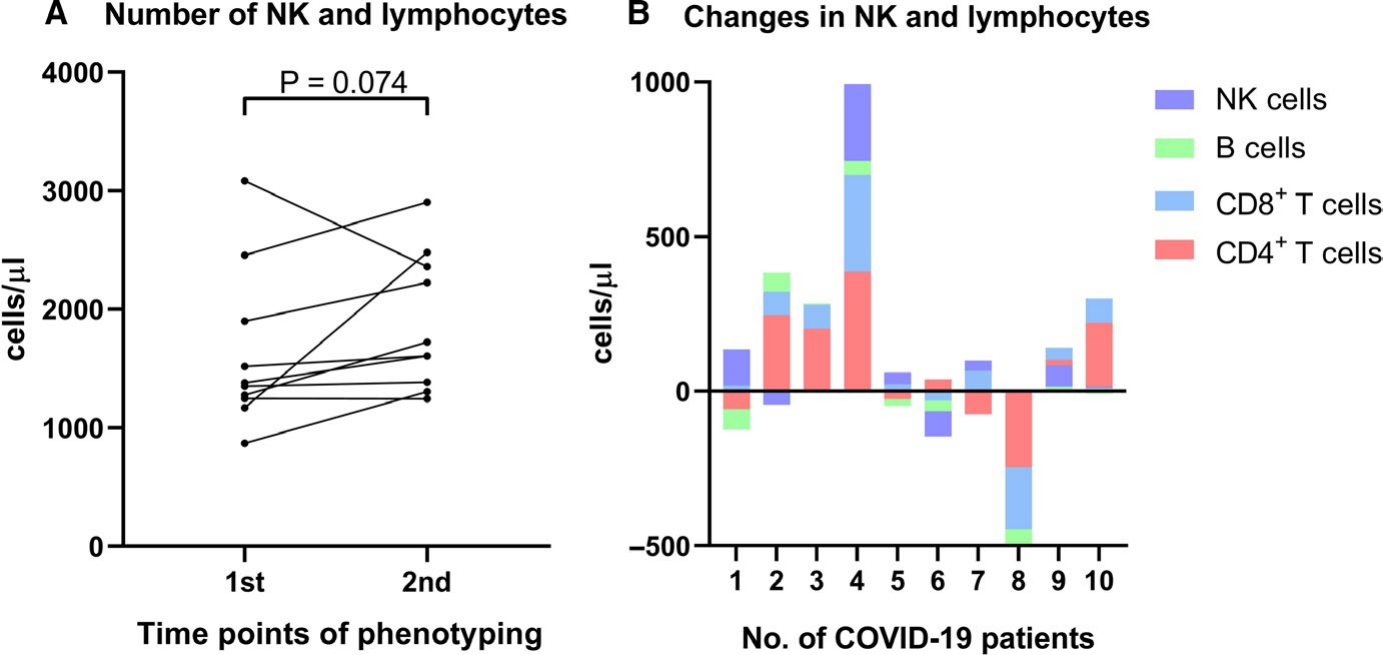
Changes in the number of NK and lymphocyte subsets in COVID‐19 patients during convalescent period. (A) Total number of NK and lymphocytes (B) Number of NK, B, CD8^+^ T and CD4^+^ T cells in patients with COVID‐19 during convalescent period. Data (A) were analysed using a paired nonparametric test. 1st: first phenotyping test on 17 February 2020; 2nd: second phenotyping test on 25 February 2020

The absolute number of the total NK cells and lymphocyte subsets in patients with COVID‐19 increased during the convalescent period (1365 cells/μL [871‐1664 cell/μL] vs 1664 cells/μL [1244‐2904 cells/μL], *P* = 0.074, Figure 3A). This increase mainly resulted from the increase in NK, CD8^+^ T and CD4^+^ T cells (Figure 3B). A decrease in B cells (Figure 3B) was observed in some cases. In summary, the number of NK cells and lymphocyte subsets increased in most patients with COVID‐19 during the convalescent period.

### 3.4 Changes in the number of CD8^+^ T, CD4^+^ T and B cell subsets in patients with COVID‐19 during convalescent period

**FIGURE 4:**
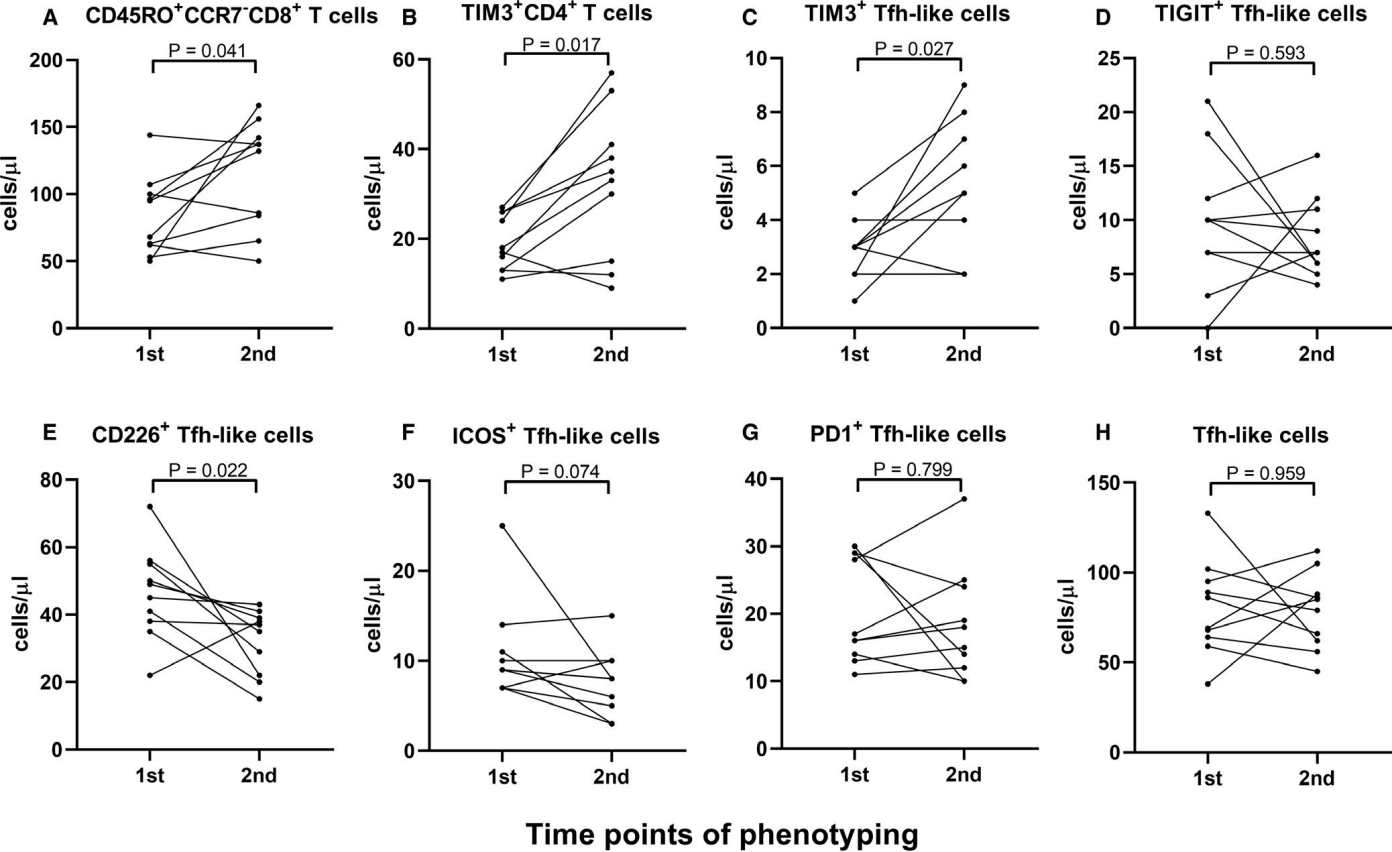
Changes in the number of multiple CD8^+^ and CD4^+^ T cell subsets in patients with COVID‐19 during convalescent period. (A) CD45^+^CCR7^−^CD8^+^ T cells (B) TIM‐3^+^CD4^+^ T cells (C) TIM‐3^+^ Tfh‐like cells (D) TIGIT^+^ Tfh‐like cells (E) CD226^+^ Tfh‐like cells (F) ICOS^+^ Tfh‐like cells (G) PD‐1^+^ Tfh‐like cells (H) Tfh‐like cells in COVID‐19 patients during convalescent period. Data were analysed using a paired nonparametric test. 1st: first phenotyping test on 17 February 2020; 2nd: second phenotyping test on 25 February 2020

The number of effector memory CD8^+^ (CD45RO^+^CCR7^−^CD8^+^) T cells in patients with COVID‐19 increased during the convalescent period (82 cells/μL [50‐144] vs 135 cells/μL [50‐166 cells/μL], *P* = 0.041, Figure 4A). The following data were observed in all patients with COVID‐19 during a convalescent period of 1 week. The absolute number of TIM3^+^CD4^+^ T cells and TIM‐3^+^ Tfh‐like cells also increased (18 cells/μL [11‐27 cell/μL] vs 34 cells/μL [9‐57 cell/μL], *P* = 0.017, Figure 4B), (3 cells/μL [1‐5 cells/μL] vs 5 cells/μL [2‐9 cells/μL], *P* = 0.027, Figure 4C). No significant difference in the absolute number of TIGIT^+^ Tfh‐like cells was observed (10 cells/μL [0‐21 cells/μL] vs 7 cells/μL [4‐16 cells/μL], *P* = 0.593, Figure 4D). The absolute number of CD226^+^ Tfh‐like cells decreased (47 cells/μL [22‐72 cells/μL] vs 36 cells/μL [15‐43 cells/μL], *P* = 0.022, Figure 4E). A decrease in the number of ICOS^+^ Tfh‐like cells was observed (9 cells/μL [7‐25 cells/μL] vs 8 cells/μL [3‐15 cells/μL], *P* = 0.074, Figure 4F). The absolute number of PD‐1^+^ Tfh‐like cells and the total number of Tfh‐like cells did not significantly change (17 cells/μL [11‐30 cells/μL] vs 17 cells/μL [10‐37 cells/μL], *P* = 0.799, Figure 4G), (78 cells/μL [38‐133 cells/μL] vs 82 cells/μL [45‐112 cells/μL], *P* = 0.959, Figure 4H). The number of B cells and B cell subsets are shown in Data S3.

**FIGURE 5:**
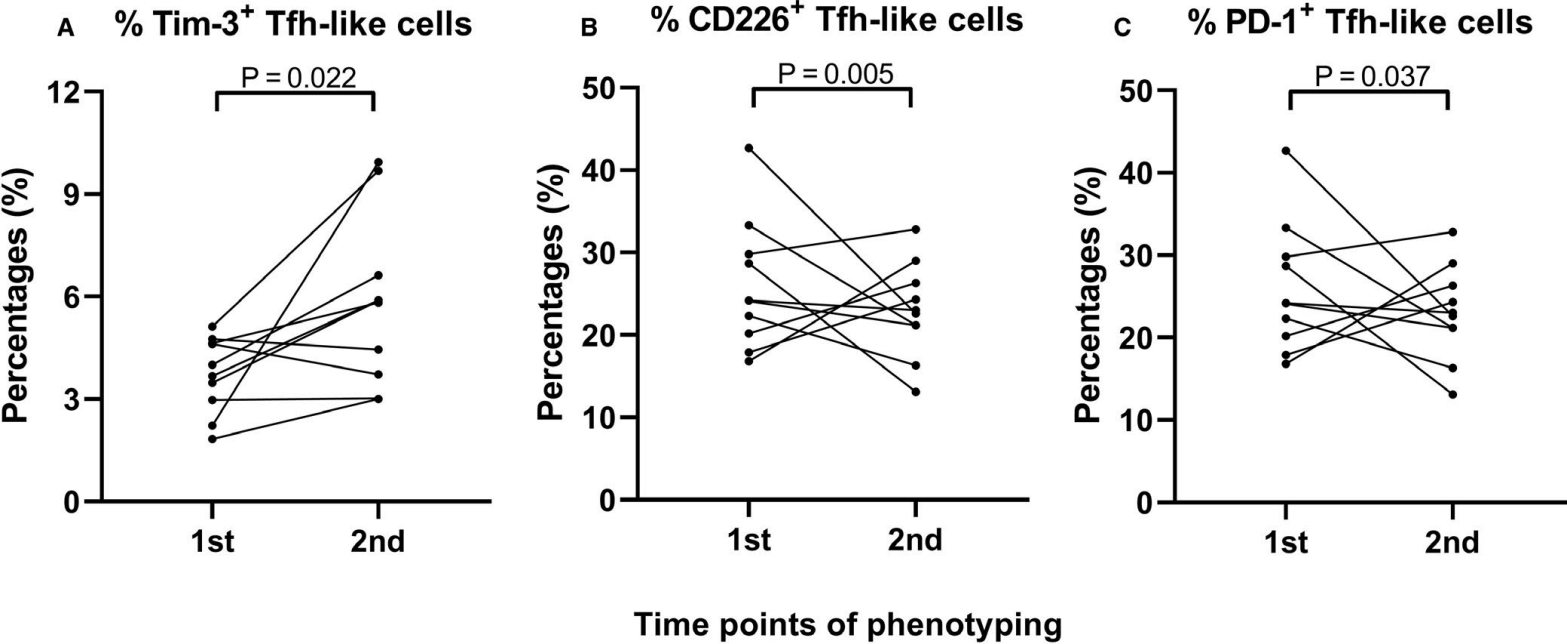
Changes in the percentage of multiple CD8^+^ and CD4^+^ T cell subsets in patients with COVID‐19 during convalescent period. (A) TIM‐3^+^ Tfh‐like cells (B) CD226^+^ Tfh‐like cells (C) PD‐1^+^ Tfh‐like cells in patients with COVID‐19 during convalescent period. Data were analysed using a paired nonparametric test. 1st: first phenotyping test on 17 February 2020; 2nd: second phenotyping test on 25 February 2020

An increase in the percentage of TIM‐3^+^ Tfh‐like cells was observed (3.84% [1.83%‐5.12%] vs 5.85% [3.00%‐9.94%], *P* = 0.022, Figure 5A). No significant difference in the percentage of TIGIT^+^ Tfh‐like cells was observed. A decrease in the percentage of CD226^+^ Tfh‐like cells was observed (58.85% [49.10%‐65.70%] vs 42.35% [34.10%‐45.70%], *P* = 0.005, Figure 5B). A decrease in the percentage of PD‐1^+^ Tfh‐like cells was observed (3.90% [2.30%‐5.60%] vs 2.26 [1.46%‐3.57%], *P* = 0.037, Figure 5C). A significant decrease in the percentage of B cells was observed (10.8% [8.0%‐19.0%] vs 9.2% [7.0%‐13.0%], *P* = 0.022). The percentage of B cell subset data are shown in Data S4. In summary, the number of effector memory CD8^+^ T cells and inhibitory CD4^+^ T cells increased in patients with COVID‐19 during the convalescent period.

### 3.5 Cytokines and inflammatory factors in patients with COVID‐19 during convalescent period

**FIGURE 6:**
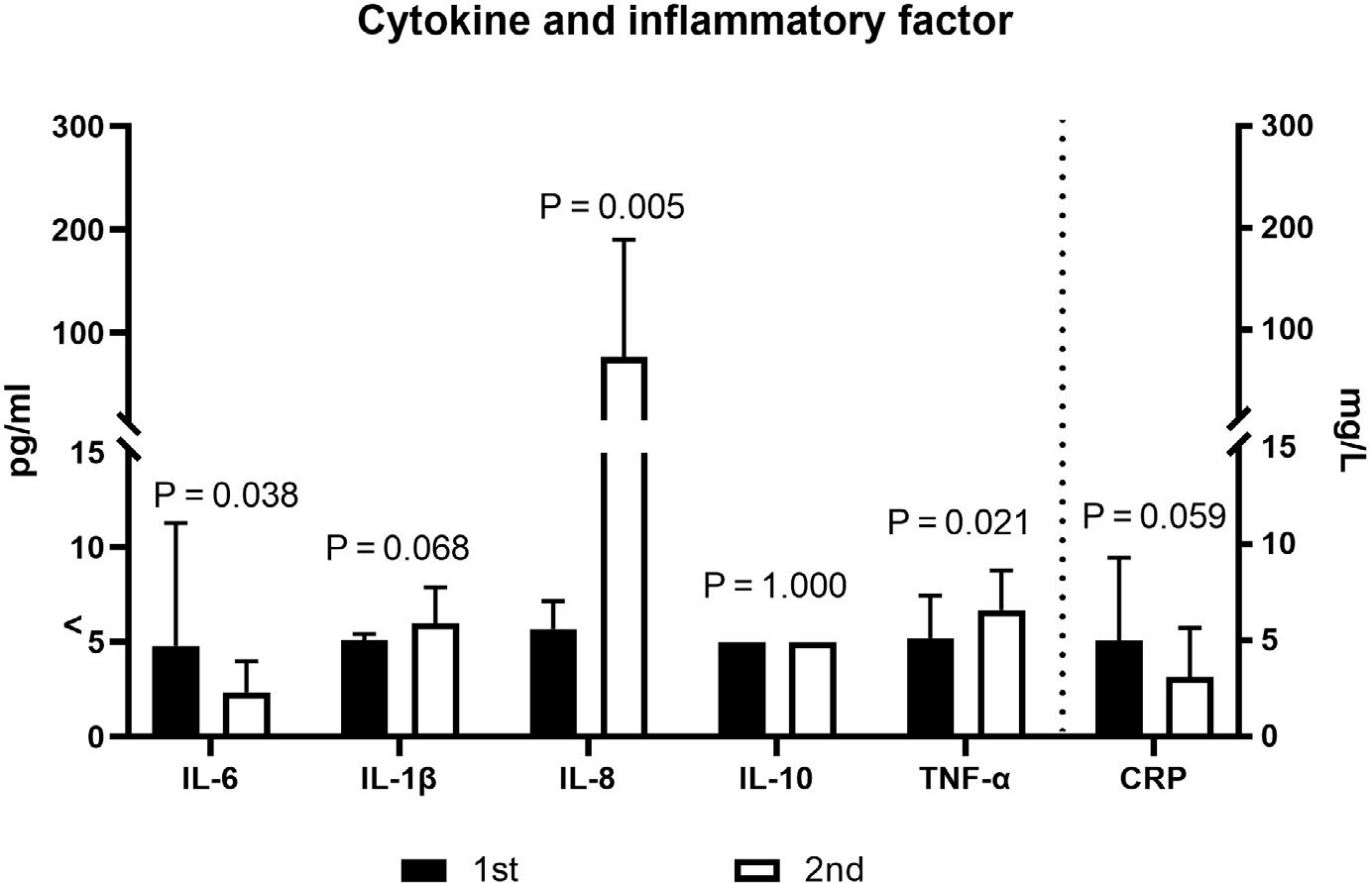
Cytokines and inflammatory factors in patients with COVID‐19 during convalescent period. Data are presented as median (95% confidence interval) and were analysed using a paired nonparametric test. 1st: first phenotyping test on 17 February 2020; 2nd: second phenotyping test on 25 February 2020

IL‐6 levels decreased significantly in patients with COVID‐19 during the convalescent period (2.33 pg/mL [<1.50‐22.40 pg/mL] vs 1.70 pg/mL [<1.50‐6.75 pg/mL], *P* = 0.038). The following data were observed in all patients with COVID‐19 during a convalescent period of 1 week. A decrease in CRP levels was observed (3.26 mg/L [<1.0‐15.0 mg/L]) vs 2.01 mg/L [<1.0‐7.75 mg/L], *P* = 0.038). IL‐8 and INF‐α levels significantly increased (<5.0 pg/mL [<5.0‐8.7 pg/mL] vs 28.7 pg/mL [8.1‐356.0 pg/mL], *P* = 0.005), (4.3 pg/mL [<4.0‐11.2 pg/mL] vs 6.7 pg/mL [<4.0‐10.6 pg/mL], *P* = 0.021). Moreover, an increase in IL‐1β levels was observed (<5.0 pg/mL [<5.0‐6.0 pg/mL] vs <5.0 pg/mL [<5.0‐10.6 pg/mL], *P* = 0.068). IL‐10 levels remained at a low level (<5.0 pg/mL). All these data are shown in Figure 6. In summary, the level of most cytokines and inflammatory factors either decreased or remained at a low level in patients with COVID‐19, during the convalescent period.

## 4 DISCUSSION

The outbreak of the highly contagious SARS‐CoV‐2 was sudden, and the various pathological damages and related pathogenesis COVID‐19 have not yet been fully understood. Many researchers have confirmed in recent studies that the immune response is a double‐edged sword in killing viruses or exacerbating immune damage.^17^, ^18^ The present study is the first to report a dynamic analysis of NK cells, CD8^+^ T lymphocytes, Tfh‐like T lymphocytes and B lymphocytes in patients with COVID‐19. In this preliminary study, we report the important role of a balanced immune response in the COVID‐19 recovery process.

In patients with moderate COVID‐19 and positive SARS‐CoV‐2‐specific antibodies, increase in the total number of NK cells and lymphocytes was observed during convalescent period. This increase resulted mainly from the increase in the number of CD4^+^ T, CD8^+^ T and NK cells. The number of B cells remained relatively stable during the same period, and the number of NK cells was significantly higher in patients with COVID‐19 than in control patients. For CD4^+^ T and CD8^+^ T cell subsets, the number of TIM‐3^+^CD4^+^ T cells, TIM‐3^+^ Tfh‐like cells and effector memory CD8^+^ T cells increased significantly during the recovery of patients. No significant difference in the number of TIGIT^+^ Tfh‐like cells was observed during disease recovery period, whereas the number of CD226^+^ Tfh‐like cells decreased significantly.

Recent studies have indicated a marked decrease in the number of peripheral lymphocytes in COVID‐19‐infected patients.^20^, ^22^ The decrease in lymphocyte counts suggested a weakened antiviral immune response. In the present study, no significant difference in the total number of NK cells and lymphocytes was observed between patients with moderate COVID‐19 and control patients (Figure 2A); the same was observed in the number of CD4^+^ T, CD8^+^ T and B cells (Figure 2C‐E). These results indicate that patients with moderate COVID‐19 exhibited an antiviral immune response similar to that in patients infected with *Mycoplasma pneumoniae* or other common pathogens. CD4^+^ T cells contribute to the infiltration of macrophages and CD8^+^ T cells into infected tissues by secreting cytokines and chemokines; they also promote the development of virus‐specific B cells and plasma cells,^10^ which emphasize the important role of T cell subsets in the pathogenesis of coronavirus infection.^10^ In addition, an increase in the number of NK, CD4^+^ T and CD8^+^ T cells was observed in most patients with moderate COVID‐19 during convalescent period (Figure 3). Hence, effective therapy can be considered to be accompanied by the enhancement of both innate and acquired immune responses.^23^ NK cells usually inhibit proliferation of viruses by stimulating the development of dendritic cells and CD8^+^ T cells via the secretion of IFN‐γ.^24^ Compared with the control patients, patients with moderate COVID‐19 had a higher number of NK cells, thereby suggesting that the activity of the innate immunity against SARS‐CoV‐2 might be stronger than other pathogens.

In a recent study, the percentages of naïve CD4^+^ T cells and memory CD4^+^ T cells were reportedly increased and decreased, respectively, in severe COVID‐19 cases.^2^ In a study by Chen et al, no difference in the percentage of CD45RA^+^CD4^+^ or CD45RO^+^CD4^+^ T cells was found between moderate and severe cases of COVID‐19.^25^ The present study focused on naïve and memory CD8^+^ T cells in patients with COVID‐19. An increased number of effector memory CD8^+^ T cells (CD45RO^+^CCR7^−^CD8^+^ T cells) was observed in moderate cases during convalescent period (Figure 4A). This result indicates that the formation of memory CD8^+^ T cells may contribute to the elimination of SARS‐CoV‐2 and improvement of clinical symptoms, but would cause neither excessive cytotoxicity nor cytokine storms. The above changes indicate that patients with moderate COVID‐19 had gradually established an antiviral immune response against secondary infection.

NK, T and B cells play a vital role in balancing the elimination of viral infection and the risk of overwhelming inflammation.^26^ Most patients with severe COVID‐19 were observed to have elevated levels of infection‐related biomarkers and inflammatory cytokines that are observed in cytokine release syndrome (CRS).^2^, ^17^, ^18^ Uncontrolled inflammatory responses may lead to local and systemic tissue damage in patients with severe COVID‐19 but not those with mild COVID‐19.^14^ During the convalescent period of moderate COVID‐19, decreased or stably low levels of most serum cytokines (IL‐6, IL‐1β, IL‐10, TNF‐α) or inflammatory factors (CRP) were observed (Figure 6). IL‐6 may promote CTL differentiation and enhance virus‐killing function. IL‐1, IL‐6, IL‐8 and TNF‐α may up‐regulate the expression of vascular endothelial adhesion molecules and chemoattract neutrophils to the site of inflammation. IL‐10, an anti‐inflammatory cytokine, is involved in the negative regulation of inflammatory response. In the present study, the low levels of serum cytokines indicated a low risk of cytokine storm or excessive immune response in moderate cases, thereby implying a good convalescent response after SARS‐CoV‐2 infection. During the same period, no marked change in the number of lymphocyte subsets was observed; this was consistent with the changes in the level of cytokines. In addition, the absolute number of TIM‐3^+^CD4^+^ T cells was significantly increased in patients with COVID‐19 during convalescent period (Figure 4B). CD4^+^ T cells are involved in antiviral responses and secrete inflammatory cytokines. Increased TIM‐3^+^CD4^+^ T cell counts may inhibit the excessive activation of CTL and NK cells, reduce the secretion of cytokines by CD4^+^ T cells and regulate immune response.^27^, ^28^, ^29^ This indicated the important role of TIM‐3 in the treatment period of SARS‐CoV‐2 infection. The increased expression of TIM‐3^+^ T cells within a certain range may help inflammatory reactions remain at a low level and prevent the occurrence of severe conditions. In a recent study, the expression of exhaustion marker NKG2A in NK cells and CD8^+^ T cells was reportedly up‐regulated in patients with COVID‐19. Upon patient recovery, the exhaustion markers on cytotoxic lymphocytes decreased.^23^ Changes in immune cell function towards memory and exhaustion stage would help prevent the risk of overwhelming inflammation and maintain proper antiviral response; these changes would eventually determine the fate of patients with COVID‐19.

Tfh cells, a subset of CD4^+^ T cells, promote antibody production by activating Tfh‐dependent B cells.^11^ The enrolled patients with COVID‐19 tested positive for SARS‐CoV‐2 antibodies; such observation was consistent with a recent finding that SARS‐CoV‐2‐neutralizing antibodies were detectable 7‐14 days after the onset of symptoms.^30^ An increase in the number of TIM‐3^+^ Tfh‐like cells in patients with COVID‐19 was observed during the convalescent period (Figure 4C). This indicated that TIM‐3 might inhibit Tfh cell function in helping B cell activation and specific antibody production. The absolute number of B cells and B‐cell subsets did not change significantly in patients with COVID‐19 during convalescent period. The inhibition of Tfh cells may contribute to the control of B cells and B‐cell subsets in patients with moderate COVID‐19. This may prevent the overproduction of specific antibodies. In previous studies, high titres of total antibodies were associated with poor outcomes of COVID‐19,^31^, ^32^ but it was unknown whether antibody responses somehow contributed to pulmonary pathology.^30^ For patients with moderate COVID‐19 and specific antibodies, an increase in the number of peripheral TIM‐3^+^ Tfh‐like cells may be a signal of the alleviation of symptoms during the treatment period and may indicate an appropriate humoural immune response.

No significant difference in the number of TIGIT^+^ or PD‐1^+^ Tfh‐like cells was observed in patients with COVID‐19 during convalescent period, whereas the number of CD226^+^ Tfh‐like cells was observed to significantly decrease during the same period (Figure 4D,E,G). This result suggests that during recovery, the ability of dendritic cells to activate Tfh cells through the CD226‐induced signalling pathway might have been weakened. CD226 may competitively inhibit the interaction between CD155 and TIGIT.^15^ The down‐regulation of CD226 may ultimately enhance TIGIT function and further inhibit Tfh cell function. Moreover, a downward trend in the activating molecule, ICOS in Tfh‐like cells, was observed (Figure 4F); this was consistent with the observation that Tfh cells shift from the activation phase to the inhibition phase during antibody production.

A recent study reported that respiratory SARS‐CoV‐2 viral load in mild patients decreased after reaching a peak during the second week after the onset of disease.^33^ In the present study, we found that the function of Tfh‐like cells in patients with moderate COVID‐19 was inhibited during the third week after the onset of COVID‐19. As viral load began to decrease, the development of Tfh‐like cells was inhibited by the up‐regulation of TIM‐3 and TIGIT (relatively) and the down‐regulation of ICOS; these processes may be correlated with therapeutic effects and stable antibody production. The number of PD‐1^+^ Tfh‐like cells did not significantly change during the convalescent period; therefore, the decrease in the percentage of PD‐1^+^ Tfh‐like cells (Figure 5C) might have resulted from the increased expression of TIM‐3 in Tfh‐like cells. During the recovery of patients with COVID‐19, the expression of PD‐1 was more stable than that of TIM‐3; this might have had prevented the excessive exhaustion of Tfh cells, thereby maintaining a balanced humoural immune response against SARS‐CoV‐2.

Strict prevention and control measures of quarantine and social distancing were observed in Sichuan Province since the outbreak of COVID‐19 from Wuhan, and only 166 confirmed cases were reported in Chengdu, China (0.19% [166/84409]).^34^ Only 11 patients with moderate COVID‐19 were hospitalized in West China Hospital when the present study was conducted; this resulted in some limitations. First, there was a lack of analysis of patients with severe cases. Second, there was a lack of analysis during the serological conversion phase, as all patients had positive virus‐specific antibodies. Third, there was a lack of data on other CD4^+^ T cell subsets (Th1, Th2, Th7 and Tregs) owing to lack of timely reagent supplies.

In conclusion, a high number of NK cells are important in anti‐SARS‐CoV‐2 immune response. The increase in the number of effector memory CD8^+^ T cells, the up‐regulation of inhibitory molecules and the down‐regulation of active molecules on CD4^+^ T cells and Tfh‐like cells would maintain balanced inflammatory responses and prevent the development of severe cases. The balanced innate, cellular and humoural immune antiviral responses and the appropriate production of antiviral antibodies are the key factors that ensure the elimination of SARS‐CoV‐2. These dynamic characteristics of immune response in patients with COVID‐19 during convalescent period may provide a scientific basis for immune response monitoring and clinical treatment strategies.

## CONFLICT OF INTEREST

The authors declare that there are no conflicts of interest.

## AUTHOR CONTRIBUTION

**Lin Yan:** Data curation (equal); Software (lead); Writing‐original draft (lead). **Bei Cai:** Resources (equal); Writing‐original draft (supporting). **Yi Li:** Formal analysis (lead); Funding acquisition (lead). **Min‐Jin Wang:** Methodology (equal). **Yun‐Fei An:** Methodology (equal). **Rong Deng:** Resources (equal). **Dong‐Dong Li:** Methodology (equal). **Li‐Chun Wang:** Conceptualization (equal); Resources (equal). **Huan Xu:** Resources (equal). **Xue‐Dan Gao:** Resources (equal). **Lan‐Lan Wang:** Funding acquisition (supporting); Project administration (lead); Supervision (lead); Writing‐review & editing (lead).

## Supporting information

Data S1‐S4

## Data Availability

The data that support the findings of this study are available from the corresponding author upon reasonable request.
