# Supplementary material for: Dynamics of NK, CD8 and Tfh cell mediated the production of cytokines and antiviral antibodies in Chinese patients with moderate COVID‐19
Source: J Cell Mol Med. 2020 Nov 3;24(24):14270–9. doi: 10.1111/jcmm.16044 (PMC7753779; doi:10.1111/jcmm.16044)
Supplement: Supplementary file 1 — Data S1‐S4 [file JCMM-24-14270-s001.docx]

**Supplementary Data 1. Number of NK and lymphocyte subsets between COVID-19 and control patients**

**Count unit：cells/μl**

**Supplementary Data 2. Percentage of NK and lymphocyte subsets between COVID-19 and control patients**

**Percentage unit：%**

**Supplementary Data 3. Changes in the number of NK and lymphocyte subsets in moderate COVID-19 during convalescent period**

**Count unit：cells/μl**

**Supplementary Data 4. Changes in the percentage of NK and lymphocyte subsets in moderate COVID-19 during the convalescent period**

**Percentage unit：%**
